# Clinical relevance of appraisals of persistent psychotic experiences in people with and without a need for care: an experimental study

**DOI:** 10.1016/S2215-0366(17)30409-1

**Published:** 2017-12

**Authors:** Emmanuelle Peters, Thomas Ward, Mike Jackson, Peter Woodruff, Craig Morgan, Philip McGuire, Philippa A Garety

**Affiliations:** a Department of Psychology, King's College London, Institute of Psychiatry, Psychology and Neuroscience, London, UK; b Health Service and Population Research, King's College London, Institute of Psychiatry, Psychology and Neuroscience, London, UK; c Psychosis Studies Department, King's College London, Institute of Psychiatry, Psychology and Neuroscience, London, UK; d South London and Maudsley NHS Foundation Trust, London, UK; e Bangor University, School of Psychology, Bangor, Gwynedd, UK; f Betsi Cadwaladr University Health Board, Bangor, Gwynedd, UK; g Sheffield Cognition and Neuroimaging Laboratory, Academic Psychiatry, University of Sheffield, Sheffield, UK; h NIHR Biomedical Research Centre for Mental Health, South London and Maudsley NHS Foundation Trust, London UK

## Abstract

**Background:**

Cognitive models of psychosis propose that appraisals (ie, the interpretation and meaning attributed to experiences) are central to the transition from anomalous experiences to psychotic symptoms. In the Unusual Experiences Enquiry (UNIQUE) study, we investigated the role of appraisals by comparing individuals with persistent psychotic experiences without a need for care with patients and people without psychotic experiences.

**Method:**

Eligible participants were patients with diagnosed psychotic disorders (clinical group) and adults in the general population with persistent psychotic experiences (non-clinical group) and without psychotic experiences (controls). The appraisals of psychotic experiences among people in the non-clinical and clinical groups were assessed by an in-depth interview, and appraisals of anomalous experiences induced by three experimental tasks were compared between all groups.

**Findings:**

We recruited 259 participants, 84 in the clinical group, 92 in the non-clinical group, and 83 controls. The clinical group was more likely than the non-clinical group to display paranoid, personalising interpretations of their psychotic experiences (p<0·008; p values are Sidak adjusted to account for multiple testing) and less likely to have normalising (p<0·008) and supernatural (p=0·039) explanations. The clinical group also appraised their psychotic experiences as being more negative, dangerous, and abnormal and less controllable than the non-clinical group (all p<0·005), but groups did not differ for attributions of general externality (p=0·44). For experimentally induced anomalous experiences, the clinical group endorsed more threatening appraisals on all tasks than the non-clinical group (p<0·003), who did not differ from the control group (p=0·07–0·6). The pattern was similar for ratings of salience, distress, personal relevance, global threat, and incorporation of the induced experiences into participants' own psychotic experiences.

**Interpretation:**

We provide robust evidence that the way psychotic experiences are appraised differs between individuals with and without a need for care, supporting cognitive models of psychosis. Specifically, the absence of paranoid and threatening appraisals might protect against persistent psychotic experiences becoming clinically relevant.

**Funding:**

UK Medical Research Council.

## Introduction

The continuity between health and psychosis is well recognised. Large-scale surveys have confirmed high prevalence of psychotic experiences in the general population,^1^ and around 20% of individuals with psychotic experiences report persistent rather than transient experiences.^2^ Although the presence of psychotic experiences is associated with an increased risk of developing psychotic disorders, for most people they do not become clinically relevant.^1^ Individuals reporting persistent, non-distressing psychotic experiences for which they have not sought help and who have never been diagnosed with a psychotic disorder (ie, do not have a need for care^1^), form a unique group of particular importance in identifying potential risk and protective factors in the development of psychosis. The Unusual Experiences Enquiry (UNIQUE) study^3^ has shown that these individuals present with hallucinations in all sensory modalities, including first-rank symptoms. Their experiences were enduring but less frequent than those in patients with psychosis, as has also been found in “healthy voice-hearers”.^4^ People without a need for care were differentiated from patients by an absence of paranoia, cognitive difficulties, and negative symptoms, which is in line with evidence that these issues are more predictive of transition to psychosis and poor functional outcomes than perceptual disturbances in ultra-high-risk individuals.^5^, ^6^ These findings, along with sociodemographic differences between the groups,^3^ support the notion that psychotic disorders arise from a complex interplay between social, environmental, psychological, and biological determinants.^7^, ^8^

According to cognitive models of psychosis, appraisals (the interpretation and meaning attributed to experiences) are central to determining whether benign psychotic experiences develop into clinically relevant psychotic symptoms.^8^, ^9^ With use of an in-depth interview approach,^10^ we have previously shown that non-clinical groups (ie, without a need for care) typically endorse normalising and spiritual or supernatural explanations of their psychotic experiences, whereas clinical groups (ie, with a need for care) are more likely to appraise their experiences as being dangerous and having been caused by other people (ie, personalising appraisals),^10^, ^11^, ^12^ which is in turn associated with distress.^13^ Importantly, the threat-based nature of the appraisals, rather than whether they were internal or external attributions, was the key differentiating factor between groups. A complementary approach to in-depth interviews is to induce anomalous experiences through experimental tasks, ensuring everyone is exposed to the same experience, and assess differences in the resultant appraisals. In pilot studies that used analogues of hearing voices and thought interference, patients scored higher on maladaptive appraisals than did non-clinical groups with persistent psychotic experiences,^14^ even when their symptoms had remitted.^15^ Therefore, the way in which psychotic experiences are interpreted, rather than merely their presence, is important to clinical status.

**Research in context**

**Evidence before this study**

Cognitive models of psychosis propose that appraisals (ie, the interpretation and meaning attributed to experiences) are central to the transition from benign psychotic experiences to psychotic symptoms. We searched PsycInfo for articles investigating appraisals of psychotic experiences published from Jan 1, 2002, to May 5, 2017, without language restrictions. Using the search term “(‘appraisals’ AND ‘psychosis’ OR ‘schizo*’ AND ‘anomalous experiences’ OR ‘psychotic experiences’ OR ‘psychotic symptoms’)”, we identified 47 studies, of which 13 were from our group. The abstracts showed that 14 were relevant experimental studies and that the remaining 33 were review papers or book chapters, therapy outcome studies, or articles focusing on schemas, metacognitive beliefs, stigma, illness beliefs, or a combination of these features, rather than appraisals. Among the 14 relevant articles, seven compared adults with psychotic experiences with and without a need for care, and we excluded seven that involved only children or adolescents, people at high risk of developing psychosis, people with high versus low schizotypy, or those with psychosis. The seven selected studies highlighted differences in appraisals related to threat between individuals with and without a need for care, meaning that they centred on attributions of danger, emotional valence, and agency, and that people with clinical diagnoses typically viewed their experiences as being caused by other people who wished them harm. The studies were, however, hampered by small sample sizes, and none provided convergent evidence on the role of appraisals obtained through both standard interviews and experimentally induced anomalous experiences.

**Added value of this study**

Our study, with a large sample size and the combined use of an in-depth interview with creative symptom-analogue tasks, showed clear and consistent differences in interpretations of individuals' own and experimentally induced psychotic experiences between those with and without a need for care. The group with a psychotic disorder were more likely to display paranoid, personalising interpretations and less likely to have normalising and supernatural explanations than the non-clinical group, and appraised their psychotic experiences as more negative, dangerous, abnormal, and less controllable.

**Implications of all the available evidence**

Our findings and those from previous pilot studies support cognitive models of psychosis that emphasise the central role of appraisals of anomalous experiences in determining the route to psychosis and need for care. The evidence suggests that not making paranoid and threatening appraisals is protective against developing problematic outcomes of persistent anomalous experiences. These findings contribute to the identification of protective factors and determinants of wellbeing in the context of psychotic experiences.

So far, studies have been hampered by small sample sizes, and none has provided convergent evidence on the role of appraisals through both standard interviews and experimentally induced anomalous experiences. The combination of these two approaches confers the advantages of providing detailed contextual information specific to the individual and the ability to assess appraisal processes in real-time under experimental conditions. We report an assessment of appraisals in a large sample of individuals with persistent psychotic experiences with and without a need for care and a control group without psychotic experiences. We tested two hypotheses: first, that those in the clinical group would be more likely than those in the non-clinical group to display paranoid and threatening appraisals and less likely to display normalising and spiritual or supernatural appraisals, but would not differ on general externality of attributions (source of experience attributed as external to the self); and, second, that clinical participants would endorse more threatening explanations of experimentally induced anomalous experiences than non-clinical participants, who in turn would not differ from the control group.

## Methods

### Study design and participants

Three groups were recruited in the UK, from urban (London) and rural (Gwynedd, north Wales) areas. The first included patients diagnosed with a psychotic disorder (the clinical group), the second individuals from the general population with persistent psychotic experiences but without a need for care (the non-clinical group), and the third people from the general population with no psychotic experiences (the control group). Potential participants were screened by research workers by telephone, or face to face if they were inpatients. We excluded people younger than 18 years, without sufficient command of English, and with histories of neurological disorders, head injury, or epilepsy, and with primary substance dependence. Further details on recruitment and groups are provided in the appendix.

Patients with positive symptoms (score ≥2 on at least one item of the Scale for the Assessment of Positive Symptoms^16^ at time of recruitment) and a clinical psychotic disorder diagnosis (ICD-10 F20–39^17^) were included in the clinical group. Participants who had received cognitive behaviour therapy for psychosis as per the National Institute for Health and Clinical Excellence guidelines (>6 months of therapy, ≥16 planned sessions, or both) were excluded because of a possible effect on appraisals.

Individuals with enduring psychotic experiences (score ≥2 on at least one item of the Scale for the Assessment of Positive Symptoms^16^ at time of recruitment) but no clinical diagnosis of or treatment for psychotic disorders formed the non-clinical group. They were recruited from specialist sources, such as online forums for psychic and spiritualist activities, mediums, and other special interests.^3^ Additionally, we recruited participants from the South East London Community Health study.^18^ The clinical and non-clinical groups did not differ significantly on overall psychotic experiences (ie, the Anomalous Experiences Interview [AANEX]-Inventory current and lifetime total scores^10^) or lifetime presence of auditory hallucinations, but there were some group differences on individual AANEX current factor scores (table 1, appendix).

**Table 1:** Demographic and clinical characteristics of the three groups

			**Control group (n=83)**	**Non-clinical group (n=92)**	**Clinical group (n=84)**	**Difference**
Site						
	London		43 (52%)	51 (55%)	43 (51%)	..
	Bangor, Gwynedd		40 (48%)	41 (45%)	41 (49%)	..
Sources			18 (22%) suggested by non-clinical participants, 65 (78%) from GP and university registers or circulars	82 (89%) specialist groups and fora, 10 (11%) from SELCoH and GP registers	29 (35%) from inpatient wards, 55 (65%) from community services	..
Sex						χ^2^=31·3, df=2, p<0·001
	Men		26 (31%)	25 (27%)	55 (66%)	..
	Women		57 (69%)	67 (73%)	29 (34%)	..
Age (years)			46 (13)	46 (14)	42 (13)	*F*_(2,256)_=2·5, p=0·09
Ethnicity						χ^2^=20·1, df=2, p<0·001 (white *vs* others)
	White		75 (90%)	80 (87%)	55 (66%)	..
	Mixed		2 (2%)	3 (3%)	4 (5%)	..
	Asian		2 (2%)	2 (2%)	2 (2%)	..
	Black		3 (4%)	6 (7%)	22 (26%)	..
	Other		1 (1%)	1 (1%)	1 (1%)	..
Education (years)			17·1 (4·0)*	16·8 (4·2)	14·7 (5·8)*	*F*_(2,254)_=6·3, p=0·002
Spiritual†			34 (41%)	82 (91%)‡	62 (77%)§	χ^2^=54·2; df=2 p<0·001
Religion						χ^2^=68·2; df=4 p<0·001
	None		48 (58%)	32 (35%)	16 (19%)	..
	Mainstream		28 (34%)	19 (21%)	55 (66%)	..
	Non-traditional		7 (8%)	41 (45%)	13 (16%)	..
IQ¶			112 (16·5)†	105 (14·0)‡	85 (14·2)‖	*F*_(2,247)_=71·1, p<0·001
Psychotic experiences						
	Age at onset (years)		..	15 (12·3)	22 (10·4)	*t*_(174)_=3·9, p<0·001
	Time since onset (years)		..	31·2 (15·3)	20·2 (12·9)	*t*_(174)_=5·1, p<0·001
Lifetime auditory hallucinations			..	71 (77%)	74 (88%)	χ^2^=3·6, df=1, p=0·06
AANEX score						
	Total lifetime**		..	34·8 (4·9)	36·3 (6·4)‡	*F*_(1,172)_=2·8, p=0· 514††
	Total current		..	28·6 (5·1)	30·1 (6·2)‡	*F*_(1,172)_=2·9, p=0· 475††
	Meaning reference factor (current)‡‡		..	7·7 (2·1)	7·5 (2·2)‡	*F*_(1,172)_=0·7, p=0· 975††
	First-rank symptoms factor (current)‡‡		..	7·5 (1·9)	8·1 (2·5)‡	*F*_(1,172)_=2·8, p=0· 507††
	Hallucinatory-paranormal factor (current)‡‡		..	5·9 (1·7)	5·1 (1·9)‡	*F*_(1,172)_=9·3, p=0·021††
	Dissociative-perceptual factor (current)‡‡		..	3·8 (1·4)	4·5 (1·8)‡	*F*_(1,172)_=7·5, p=0·048††
	Cognitive-attentional factor (current)‡‡		..	3·8 (1·6)	5·1 (1·7)‡	*F*_(1,172)_=28·4, p<0·007††
Diagnosis (ICD-10)						
	Schizophrenia		..	..	53 (63%)	..
	Schizoaffective		..	..	13 (16%)	..
	Psychosis NOS		..	..	6 (7%)	..
	F30–39		..	..	11 (13%)	..
Antipsychotic medications and doses						
	None		..	..	8 (10%)	..
	Medicated		..	..	76 (90%)	..
		Typical	..	..	8 (10%)	..
		Atypical	..	..	47 (62%)	..
		Clozapine	..	..	21 (28%)	..
		>1 antipsychotic	..	..	13 (17%)	..
		Additional psychotropic medications	..	..	55 (72%)	..
		Median percentage maximum daily recommended dose (range)	..	..	50% (12–100)§§	..
Hospital admissions			..	..	4·4 (3·6)¶¶	..

Data are number (%) or mean (SD). GP=general practitioner. SELCoH=South East London Community Health Study.^18^ AANEX=Appraisals of Anomalous Experiences Interview-Inventory.^10^

* One participant missing.

† Answered ‘Yes’ to “Would you describe yourself as a spiritual person”.

‡ Two participants missing.

§ Three participants missing.

¶ Estimated with four subtests of the Wechsler Adult Intelligence Scale, third edition—Short Form (WAIS-III):^21^ information, block design, arithmetic, and digit symbol.

‖ Six missing participants.

** Potential range of scores for both totals 17–51.

†† p value Sidak adjusted for seven multiple tests.

‡‡ Factor scores range from 3 to 9 (4–12 for meaning reference and first-rank symptoms).

§§ Four participants missing.

¶¶ Five participants missing.

The control group comprised individuals who endorsed no items on the Unusual Experiences Screening Questionnaire, which is derived from the AANEX-Inventory section^10^ and the Psychosis Screening Questionnaire,^19^ and scores of 1 or less SD from the unusual experiences subscale mean of the Oxford-Liverpool Inventory of Feelings and Experiences.^20^ They were recruited from community settings or volunteered by non-clinical participants.

The London-Westminster National Research Ethics Service Committee (12/LO/0766), South London and Maudsley National Health Service Foundation Trust/Institute of Psychiatry, Psychology and Neuroscience Research and Development (R&D2012/047), and Betsi Cadwaladr University Health Board R&D (Jackson/LO/0766) approved the study. All participants provided written informed consent before they were enrolled in the study.

### In-depth interview

Complete screening, cognitive assessment, and clinical measures used to characterise the groups are provided in the appendix. The key measure for this study was AANEX,^10^ which was administered to the clinical and non-clinical groups, but not the controls. Part one of the interview (AANEX-Inventory; appendix), which consists of 17 psychotic experiences each rated for presence and severity in the person's lifetime and the previous 1 month, was used to generate factor scores for meaning reference, first-rank symptoms, hallucinatory-paranormal, dissociative-perceptual, and cognitive-attentional psychotic experiences (table 1) via summation of individual item scores. Part two of the interview (AANEX-Context, Appraisal, and Response [AANEX-CAR]) was used to cover emotional and cognitive factors associated with these psychotic experiences (table 2). Participants were asked “How do you make sense of your experiences?” to elicit appraisals, which were classified in eight appraisal categories (biological, drug-related, spiritual, other people, psychological, no interpretation, supernatural, and normalising) and rated by the interviewer as being present (score of 2), possibly present (1), or not present (0). The categories were not mutually exclusive, therefore participants could be assigned ratings in more than one category. Five dimensions of appraisal were also rated by interviewers, on a scale of 1–5: valence, threat, externality, agency, and abnormality. One further dimension, controllability, was self-rated by participants, also on a scale of 1–5.

**Table 2:** Inter-rater reliability and comparison of clinical and non-clinical groups for AANEX-CAR appraisals

	**Inter-rater reliability (n=35)**		Regression analyses*			
	κ (SE)	Percentage agreement (%)	Clinical (n=81)	Non-clinical (n=92)	Odds ratio (95% CI)	p value†
**Appraisal categories**						
Biological	0·89 (0·15)	92·5%	38·6%	9·8%	4·21 (1·72–10·27)	0·016
Drug-related	0·77 (0·17)‡	94·3%	15·7%	1·1%	10·45 (1·26–86·50)	0·216
Spiritual	0·68 (0·16)	83·9%	43·4%	65·2%	0·51 (0·26–1·02)	0·380
Other people	0·72 (0·16)	91·4%	45·8%	7·6%	10·13 (3·79–27·09)	<0·008
Psychological	0·58 (0·14)	83·9%	34·9%	21·7%	1·28 (0·60–2·75)	0·997
No interpretation	NC	NC	8·4%	15·2%	0·33 (0·10–1·08)	0·426
Supernatural	0·76 (0·16)	87·7%	34·9%	67·4%	0·37 (0·18– 0·74)	0·039
Normalising	0·96 (0·15)	98·1%	26·5%	80·4%	0·09 (0·04–0·21)	<0·008
**Appraisal dimensions**						
Valence	0·79 (0·12)	88·0%	4 (3–5)	1 (1–2)	0·05 (0·05–0·11)	<0·005
Threat	0·88 (0·12)	93·0%	4 (2–5)	1 (1–2)	0·07 (0·04–0·15)	<0·005
Externality	0·77 (0·12)	87·3%	3 (2–5)	3 (3–4)	0·60 (0·32–1·12)	0·442
Abnormality	0·88 (0·12)	95·0%	4 (2–5)	1 (1–2)	0·11 (0·05–0·22)	<0·005
Controllability§			2 (1–3)	4 (2–4)	2·92 (1·57–5·43)	0·005
Agency¶	0·38 (0·12)	67·1%	..	..	..	..

AANEX=Appraisals of Anomalous Experiences Intervew. AANEX-CAR=AANEX-Context, Appraisal, and Response. NC=not calculated because all scores except one were zeros.

* Controlled for the three AANEX factors on which the groups differed and with site included as a covariate; percentages represent proportion present; scores for appraisal dimensions are medians (IQRs) and are scaled as follows: valence 1=strongly positive, 3=balance of positive and negative or neutral, 5=strongly negative; threat 1=completely harmless, 3=balanced or neutral, 5=definitely dangerous or harmful; externality 1=entirely due to internal factors, 3=balanced, 5=entirely external to self; abnormality 1=completely normal, 3=balanced, 5=completely abnormal; controllability 1=none, 3=some, 5=total; and agency 1=source entirely impersonal, 3=balanced, 5=entirely personal.

† All Sidak-adjusted values: appraisal categories adjusted for eight multiple tests; appraisal dimensions adjusted for five multiple tests.

‡ Non-weighted κ calculated because scores fell into two categories only.

§ Self-rated (ie, no inter-rater reliability calculated).

¶ Dropped from analyses owing to poor inter-rater reliability.

Interviews were audio recorded with the participant's consent. Inter-rater reliability for the eight categories and five dimensions of appraisals was good except for the agency dimension, which was dropped from further analyses (table 2, appendix).

### Anomalous experience tasks

As an analogue of thought interference symptoms,^14^ we used The Clifford Pickover ESP Experiment, commonly known as the cards task. This task gives the impression that a computer has read the participant's mind. Participants are shown six “face” playing cards on a computer screen and asked to memorise one. The participant is informed that the card will be selected and removed in a subsequent display of cards. The screen image is replaced with five cards for 3 s. The task relies on people scanning for the card they have chosen and not noticing that all cards have been replaced with slightly different ones.

As a further analogue of thought interference, we used the Telepath smartphone application.^15^ Four similar items (numbers in this study) are presented to a participant on a smartphone screen and he or she is asked to choose one before placing the smartphone screen down and revealing his or her choice to the experimenter. Unknown to participants, the movement of putting the phone down activates the application to scroll through all four items consecutively in an animation, with each transition being signalled by a sound that enables the experimenter to keep track of the number of changes. At the appropriate time, the experimenter lifts the smartphone, which freezes the animation. Thus, the experimenter seems to have chosen the correct item by “mindreading”.

We used the Virtual Acoustic Space Paradigm (VASP) as an analogue of auditory hallucinations.^14^ Acoustic manipulation via computer software causes sounds heard via headphones to be perceived as being located inside or outside the head.^14^, ^15^ Participants are told that the task assesses the effects of distraction on performance, and are asked to determine the presence of objects in blurred images while wearing headphones. Throughout the task the participant hears white noise (heard inside the head) with his or her own name (recorded by the participant before the start of the experiment) followed by the command “listen up” superimposed at random times (heard outside the head).

After each task, spontaneous explanations for the anomalous experiences were elicited from participants to find out whether they had guessed the manipulation correctly. Subsequently, they were asked to rate how much they believed various prespecified possible explanations to be true (from 0 not at all to 10 totally; panel). The choices reflected the most relevant appraisal styles, as ascertained in previous studies:^10^, ^14^ normalising, personalising, intentionalising, generalising, and externalising or internalising. The explanations were classified as threatening (n=5) or non-threatening appraisals (n=2). Global dimensions related to salience, distress, and threat elicited by the task were rated on scales of 0–10. Personal relevance and incorporation into their own psychotic experiences (yes or no answers) were also assessed (panel). At the end of the study, participants were debriefed and were given an honorarium of £30.

Panel **Appraisal styles and dimensions in the anomalous experience experimental tasks**

**Non-threatening appraisals**

*External, normalising*

• Card task: “It is just a simple card puzzle.”
• Telepath application task: “It is just a simple number puzzle.”
• VASP task: “It is part of the study and involves a pre-recorded voice.”

*Internal, normalising*

• All tasks: “It is because of the way the human mind works, just part of normal human experience.”

**Threatening appraisals**

*External, personalising*

• Card task: “It is not the computer which guessed; there is someone involved in this.”
• Telepath application task: “It was not just about this phone; there is someone behind the scenes involved in this.”
• VASP task: “Someone was speaking to me.”

*External, non-personalising*

• Card and Telepath application task: “It works because the system is able to read people's minds.”
• VASP task: “There was a spirit or some kind of entity in the room.”

*External, intentionalising*

• All tasks: “It was done on purpose to trick me or make me look stupid.”

*External, generalising*

• All tasks: “It is a trick that is part of a bigger conspiracy.”

*Internal, non-normalising*

• All tasks: “This means that something is wrong with me.”

**Global dimensions**

*Salience*

• “How striking/unusual did you find the experience?”

*Distress*

• “How distressing did you find the experience?”

*Threatening*

• “How threatening did you find the experience?”

*Personal relevance*

• “It works the same with everybody” versus “It is something specific to me.”

*Incorporation*

• “Is what happened in the task part of your ongoing experiences?”

VASP=Virtual Acoustic Space Paradigm.

### Statistical analysis

Mean ratings were calculated for threatening and non-threatening appraisals per task. Appraisal ratings were not normally distributed and, therefore, we used the Kruskal-Wallis test to test for significant differences (p<0·05) between the groups, followed by the Mann-Whitney *U* test to assess the differences between individual groups where appropriate. We also report effect sizes (*r*) for these comparisons (0·1 small, 0·3 medium, and 0·5 large). Categorical variables were analysed with the χ^2^ test or, if the expected value in cells was less than 5, Fisher's exact test. We present Sidak-adjusted p values throughout to account for multiple testing. These are calculated as p_Sidak_=1 – (1 – unadjusted p)^n^, where n is the number of multiple tests.

Since there were few ratings of 1 (“possibly present”) on the AANEX-CAR appraisals categories (range 1–11%), we recoded answers as a binary variable (no *vs* perhaps or yes). We used binary logistic and ordinal regressions to test for group differences on the appraisal categories and dimensions, respectively, controlling for AANEX inventory factor scores on which the groups differed (cognitive-attentional, dissociative-perceptual, and paranormal-hallucinatory). In the logistic regressions we entered group (non-clinical group as the reference category) into block 1 to assess its independent effect on appraisal categories and factor score into block 2. For the ordinal regressions, group was entered as the independent variable, appraisal dimensions as the dependent variable, and factor scores as covariates. Results presented for the logistic regressions are odds ratios (ORs) for block 2 unless specified otherwise, and include the covariates for the ordinal regressions.

The non-clinical group differed from the clinical group on several demographic variables (appendix), as is typical for these samples.^3^, ^10^, ^14^ Controlling for differences inherent to group status is inappropriate^22^ and, therefore, we tested for group differences on our hypothesised variables without including established risk factors for psychosis (eg, IQ, ethnicity, and sex) or factors inherent to need-for-care status (eg, education, impaired functioning, anxiety, and depression) as covariates.^14^, ^15^, ^23^

We tested for differences between sites for all task measures (Mann-Whitney *U* or χ^2^ tests) and AANEX appraisal categories (binary logistic regressions) and dimensions (ordinal regressions). None was significant, apart from the AANEX category of “no interpretation” (p=0·032 adjusted for eight multiple tests). Site was included as a covariate (block 3) for this analysis only. We did all analyses with SPSS version 24.

### Role of the funding source

The funders had no role in the study design, data collection, data analysis, data interpretation, or writing of the report. The corresponding author had full access to all the data in the study and had final responsibility for the decision to submit for publication.

## Results

We recruited 84 individuals to the clinical group, 92 to the non-clinical group, and 83 to the control group. The demographic and clinical characteristics of all participants are presented in table 1.

As predicted, the clinical group was significantly more likely than the non-clinical group to make “other people” appraisals and less likely to make “normalising” and “supernatural” appraisals of their psychotic experiences on the AANEX-CAR (table 2). Additionally, participants in the clinical group were more likely to make biological appraisals and to rate their psychotic experiences as more negative, more dangerous, more abnormal, and less controllable than those in the non-clinical group, but attributions of general externality did not differ.

The likelihood of making spiritual appraisals was significantly lower in the clinical than in the non-clinical group (OR 0·41, 95% CI 0·22–0·75, p=0·032), but this association became non-significant (p=0·38) after adjustment for AANEX-Inventory factor scores.

The tasks showed good face validity for inducing anomalous experiences, being rated as moderately striking without being unduly distressing (n=254; cards task mean salience 4·63 [SD 3·20] and distress 0·66 [1·71]; Telepath application task salience 4·49 [3·69] and distress 0·60 [1·55]; and VASP task salience 4·09 [3·18] and distress 1·85 [2·81]). Most participants did not guess the true nature of the tasks for the cards task (clinical group 95%, non-clinical group 86%, and control group 84%) or Telepath application task (99%, 93%, and 95%). Fewer did not guess the true nature of the VASP task (86%, 82%, and 61%), although it was rated as equally striking as and slightly more distressing than the other two tasks. The proportion of participants who guessed correctly the true nature of the tasks did not differ for the cards task (χ^2^=5·5, df 2, p=0·95) or Telepath application task (χ^2^=3·2, df 2, p=0·51). A difference was, however, seen for the VASP task (χ^2^=16·8, df 2, p<0·003), which was driven by more correct guesses in the control group than in the clinical (χ^2^=13·2, df 1, p<0·003) and the non-clinical (χ^2^=9·6, df 1, p=0·006) groups; the clinical and non-clinical groups did not differ from each other (χ^2^=0·52, df 1, p=0·85; all p values adjusted for three multiple tests).

The three groups were compared on their appraisals of the experimentally induced anomalous experiences on the three tasks. Sensitivity analyses showed that group differences for appraisal ratings were not affected by the inclusion or exclusion of correct guesses on any of the tasks and, therefore, all participants were included in the following analyses. As predicted, there were highly significant group differences for threatening appraisal ratings on all anomalous experiences tasks, with the clinical group scoring higher than the non-clinical group (with high effect sizes, range *r*=0·51–0·54) and the control group (with medium to high effect sizes, *r*=0·41–0·46, table 3). The clinical group had lower ratings than the non-clinical and control groups for non-threatening appraisals in the cards task, but not in the other two tasks (table 3). The participants in the clinical group also rated the tasks as more striking, distressing, and globally threatening than the other two groups (table 3). They were more likely to think the experimentally induced anomalous experiences were specific to them than the non-clinical and control groups, and more likely than the non-clinical group to incorporate them into their own psychotic experiences (table 3). The control and non-clinical groups did not differ from each other on any measure, apart from the non-clinical group rating the Telepath application task as less striking than the control group (table 3).

**Table 3:** Comparisons of threatening versus non-threatening appraisal scores, global ratings, personal relevance, and incorporation

	Clinical(n=84)*	**Non-clinical (n=92)**†	**Controls (n=83)**‡	**Effect size (2 df)**§	**Clinical vs non-clinical (1 df)**	**Non-clinical *vs* controls (1 df)**	**Clinical *vs* controls (1 df)**
**Threatening appraisals**							
Cards task	2·45 (2·54)	0·40 (0·78)	0·55 (0·78)	K=52·80, p<0·006	*U*=1616, p<0·003, *r*=0·52	*U*=3115, p=0·070, *r*=0·17	*U*=1769, p<0·003, *r*=0·41
Telepath application task	2·05 (1·97)	0·32 (0·65)	0·54 (0·82)	K=58·58, p<0·006	*U*=1537, p<0·003, *r*=0·54	*U*=3173, p=0·121, *r*=0·15	*U*=1772, p<0·003, *r*=0·44
VASP task	1·52 (1·86)	0·20 (0·55)	0·23 (0·53)	K=56·84, p<0·006	*U*=1714, p<0·003, *r*=0·51	*U*=3542, p=0·595, *r*=0·09	*U*=1663, p<0·003, *r*=0·46
**Non-threatening appraisals**							
Cards task	5·06 (2·91)	6·16 (2·76)	6·25 (2·67)	K=8·64, p=0·076	*U*=2898, p=0·047, *r*=0·18	*U*=3692, p=0·993, *r*=0·02	*U*=2477, p=0·021, *r*=0·21
Telepath application task	4·73 (3·17)	5·56 (3·13)	5·25 (3·06)	K=3·44, p=0·696	*U*=3080, p=0·169, *r*=0·14	*U*=3475, p=0·882, *r*=0·05	*U*=3045, p=0·627, *r*=0·09
VASP task	5·91 (2·59)	6·64 (2·42)	6·55 (2·27)	K=2·20, p=0·910	*U*=3151, p=0·407, *r*=0·11	*U*=3722, p=0·986, *r*=0·02	*U*=2917, p=0·611, *r*=0·09
**Global salience**							
Cards task	6·28 (3·09)	3·61 (3·00)	4·17 (2·90)	K=31·68, p<0·015	*U*=1978, p<0·003, *r*=0·40,	*U*=3338, p=0·469, *r*=0·10	*U*=2019, p<0·003, *r*=0·34
Telepath application task	6·16 (3·43)	3·22 (3·15)	4·20 (2·85)	K=31·72, p<0·015	*U*=1993, p<0·003, *r*=0·40	*U*=2900, p=0·041, *r*=0·19	*U*=2215, p<0·003, *r*=0·31
VASP task	5·38 (3·25)	3·46 (3·14)	3·57 (2·81)	K=17·25, p<0·015	*U*=2455 p<0·003, *r*=0·28	*U*=3621, p=0·909, *r*=0·05	*U*=2248, p<0·003, *r*=0·27
**Global distress**							
Cards task	1·70 (2·61)	0·22 (0·80)	0·13 (0·44)	K=42·23, p<0·015	*U*=2394, p<0·003, *r*=0·40	*U*=3751, p=0·999, *r*=0·01	*U*=2114, p<0·003, *r*=0·40
Telepath application task	1·34 (2·25)	0·26 (0·79)	0·24 (0·96)	K=25·76, p<0·015	*U*=2700, p<0·003, *r*=0·30	*U*=3592, p=0·920, *r*=0·04	*U*=2445, p<0·003, *r*=0·33
VASP task	3·08 (3·35)	1·36 (2·46)	1·24 (2·22)	K=19·96, p<0·015	*U*=2494, p<0·003, *r*=0·29	*U*=3813, p=1·0, *r*<0·01	*U*=2205, p<0·003, *r*=0·30
**Global threat**							
Cards task	1·69 (2·79)	0·16 (0·60)	0·10 (0·37)	K=43·96, p<0·015	*U*=2421, p<0·003, *r*=0·40	*U*=3712, p=0·973, *r*=0·03	*U*=2100, p<0·003, *r*=0·41
Telepath application task	1·22 (2·13)	0·19 (0·81)	0·13 (0·49)	K=31·31, p<0·015	*U*=2618, p<0·003, *r*=0·34	*U*=3678, p=0·999, *r*=0·01	*U*=2433, p<0·003, *r*=0·34
VASP task	2·08 (3·03)	0·95 (2·04)	0·82 (1·78)	K=9·55 p=0·114	*U*=2924, p=0·030, *r*=0·20	*U*=3779, p=0·998, *r*=0·01	*U*=2589, p=0·021, *r*=0·21
**Personal relevance**							
Cards task	Yes 13 (16%) No 66 (84%)	Yes 3 (3%) No 89 (97%)	Yes 1 (1%) No 80 (99%)	χ^2^=15·30, p<0·015	χ^2^=8·73, p=0·012, OR 5·84 (95% CI 1·60–21·34)	χ^2^=0·78, p=0·945, OR 2·70 (95% CI 0·28–26·45)	χ^2^=11·61, p=0·003 OR, 15·76 (95% CI 2·01–123·62)
Telepath application task	Yes 13 (16%) No 69 (84%)	Yes 4 (4%)) No 86 (96%)	Yes 1 (1%)) No 82 (99%)	χ^2^=13·65, p=0·015	χ^2^=6·27, p=0·056, OR 4·05 (95% CI 1·26–12·98)	χ^2^=1·62, p=0·750, OR 3·81 (95% CI 0·42–34·84)	χ^2^=11·40, p=0·003, OR 15·45 (95% CI 1·97–121·09)
VASP task	Yes 21 (28%) No 55 (72%)	Yes 3 (3%) No 89 (97%)	Yes 2 (2%) No 81 (98%)	χ^2^=30·68, p<0·0015	χ^2^=20·19, p<0·003, OR 11·33 (95% CI 3·23–39·76)	χ^2^=0·11, p=1·0, OR 1·37 (95% CI 0·22–8·38)	χ^2^=20·40, p<0·003, OR 15·46 (95% CI 3·48–68·63)
**Incorporation**							
Cards task	Yes 30 (37%) No 50 (63%)	Yes 4 (4%) No 88 (96%)	..	..	χ^2^=29·65, p<0·0015, OR 13·2 (95% CI 4·40–39·64)	..	..
Telepath application task	Yes 32 (40%) No 49 (60%)	Yes 5 (6%) No 84 (94%)	..	..	χ^2^=29·12, p<0·0015, OR 11·20 (95% CI 4·09–30·66)	..	..
VASP task	Yes 26 (34%) No 51 (66%)	Yes 6 (6%) No 86 (94%)	..	..	χ^2^=20·27, p<0·0015, OR 7·31 (95% CI 2·82–18·95)	..	..

Data for groups are mean (SD) or number (%). All p values are Sidak adjusted for six multiple tests for threatening and non-threatening appraisal ratings, 15 for global ratings, personal relevance, and incorporation, and three for individual group comparisons. K=Kruskal-Wallis. *U*=Mann-Whitney *U*. VASP=Virtual Acoustic Space Paradigm. OR=odds ratio.

* Four participants had missing data for the cards task (five on personal relevance), two for the Telepath application task (three on incorporation), and five for the VASP task (six on threatening and non-threatening appraisals, seven on incorporation, eight on personal significance).

† Two participants had missing data for the Telepath application task (three on global dimensions and incorporation).

‡ One participant had missing data for the cards task (two on personal relevance), and one for the Telepath application task (one on non-threatening appraisals).

§ Effect sizes for Mann-Whitney *U* values calculated as *r*=Z / square root of N, where N is the number of samples; 0·1 indicates small effect, 0·3 medium, and 0·5 large.

To minimise the number of analyses, we did not analyse individual appraisal ratings, but have illustrated these in the figure. These ratings clearly show a consistent pattern for all individual threatening appraisals across the three tasks.

**Figure:**
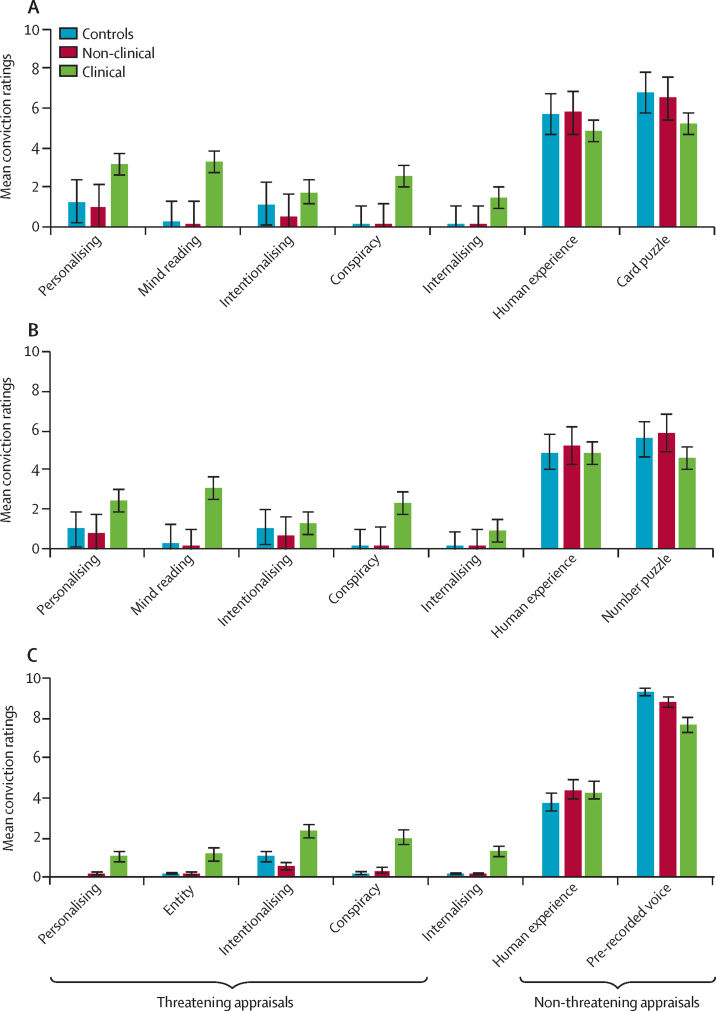
Individual appraisals in the three anomalous experience tasks Data are mean (SE). (A) Card task (control group n=82, non-clinical group n=92, and clinical group n=80). (B) Telepath application task (control group n=83, non-clinical group n=90, and clinical group n=82). (C) Virtual Acoustic Space Paradigm task (control group n=83, non-clinical group n=92, and clinical group n=78).

## Discussion

With an in-depth interview and three experimental tasks that induced anomalous experiences, we found that appraisals differentiate individuals reporting persistent psychotic experiences with and without a need for care. The clinical group was most likely to report paranoid, personalising interpretations of their psychotic experiences on AANEX and least likely to have normalising and supernatural explanations. Participants in this group also appraised their psychotic experiences as more negative, dangerous, abnormal, and less controllable than the non-clinical group. These results were mirrored by those obtained for experimentally induced anomalous experiences. The clinical group had higher conviction scores on a range of prespecified threatening appraisals than the non-clinical group, who did not differ from controls without psychotic experiences. The clinical group also rated the induced anomalous experiences as more striking, distressing, globally threatening, personally relevant, and likely to be related to their own psychotic experiences than the non-clinical group, who again did not differ from controls. Our findings support our hypotheses, replicate those of previous pilot studies,^14^, ^15^ and support cognitive models^8^, ^9^ that emphasise the central role of maladaptive appraisals of anomalous experiences in psychosis and need for care.

We also replicated previous findings that external appraisals (not recognising that your psychotic experiences are a product of your own mind) per se do not differentiate clinical from non-clinical groups, which was the only dimension on which the groups did not differ. Indeed, the non-clinical group was more likely to express specific types of external appraisals, such as supernatural explanations, whereas the clinical group was more likely to make internal attributions, such as biological explanations, for their own psychotic experiences, and non-normalising internal appraisals for the anomalous experience tasks (“this means that something is wrong with me”). Participants in the clinical group were also more likely to identify their psychotic experiences as abnormal than their non-clinical counterparts, further suggesting that insight into psychotic experiences is not a discriminating factor. Rather, a central issue differentiating benign from pathological outcomes of psychotic experiences is whether appraisals indicate a threat to self and, specifically, whether they involve the malevolent intent of other people. These results fit with previous findings that individuals without a need for care generally do not present with persecutory delusional beliefs,^3^ and suggest that a paranoid world view might be central to other types of distressing psychotic symptoms. Cognitive models of positive symptoms need to be updated to incorporate the consistent finding that threat-based, rather than external, appraisals are central to distinguishing benign anomalous experience from distressing psychotic symptoms.^24^

As expected, the non-clinical group was more likely to display spiritual appraisals than the clinical group, but this association was weakened after adjustment for the psychotic experiences on which the two groups differed (appendix).^3^ A similar pattern was reported by Brett and colleagues,^10^ which suggests that non-clinical individuals might be more likely than clinical individuals to have the kinds of experiences that elicit spiritual explanations. For instance, the non-clinical group scored higher than the clinical group on the AANEX paranormal-hallucinatory factor, which includes experiences such as precognition and perception of other entities or energies. These experiences might be more easily interpreted as being caused by spiritual agents than thought blockages (ie, the cognitive-attentional factor, on which the non-clinical group scored lower than the clinical group). Therefore, our findings show that psychotic experiences are difficult to disentangle from their interpretations; variation in the content of psychotic experiences might affect the likelihood of eliciting spiritual or benevolent appraisals, which in turn could shape their phenomenology.

The use of tasks allowed us to apply experimental control over the anomalous experiences, avoiding variation in content of experiences across groups, although this strategy has other limitations. All groups rated the non-threatening appraisals as most likely overall, as would be expected from the deliberately mild nature of the anomalous experiences. Nevertheless, ratings for threatening appraisals were higher on all three tasks for the clinical than the non-clinical group, and clinical participants were more likely to report that the task-induced anomalous experience was specific to them and related to their own psychotic experiences. Therefore, even apparently innocuous experiences might sow the seeds of paranoid thoughts and be incorporated into ongoing psychotic experiences in clinical individuals.

Our non-clinical group was a highly selected sample of high-functioning individuals, many of whom were members of subcultural groups that provide validation and acceptance of their psychotic experiences. Therefore, they might not be representative of people with psychotic experiences in the general population, who have been shown in other studies to have possible unmet mental health needs.^25^ Our aim, however, was not to characterise a general population sample with psychotic experiences, but to compare individuals with poor and good outcomes of psychotic experiences, and our results are informative within this context. Our clinical and non-clinical groups also differed in several demographic variables, which is typical for these samples.^3^, ^10^, ^14^ We deliberately did not control for these differences because we deemed them to be established risk factors for psychosis and inherent to need-for-care status rather than confounding variables. These factors are likely to drive the groups' appraisals of their psychotic experiences and their environments, as would be predicted by biopsychosocial models of psychosis.^7^, ^8^

A substantial minority of participants guessed the experimental manipulation in the VASP task, which might have compromised its validity as an analogue of auditory hallucinations.^14^ Nevertheless, the pattern of the results for this and the other tasks was identical, which suggests that it elicited an anomalous experience. Lastly, we cannot determine from our findings whether appraisals are causally implicated in the route to psychosis, although the study design has the benefit of showing differences in appraisals between groups under controlled conditions.

Our findings have implications for psychiatric practice. Since attributions to external, albeit benevolent, causes, such as spiritual guidance or supernatural entities, were highly prevalent in the non-clinical group, the biological or psychological explanations offered by mental health services might not necessarily be the most adaptive interpretations of psychotic experiences. Clinicians can be accepting of a range of interpretations to promote recovery without being overly concerned about colluding, and recognise that a focus on eradication of psychotic experiences, development of biomedical-based insightful understanding, or both, might be less helpful than one that, instead, considers threat, salience, and emotional valence as key targets. Psychological therapies,^26^, ^27^ specifically, already have a normalising and accepting approach to psychotic experiences as a central tenet,^28^ and need to prioritise the tackling of appraisals that are a threat to self-esteem^29^ and the reduction of cognitive biases that predispose individuals to process information in a threatening manner.^30^

For more on the **The Clifford Pickover ESP Experiment** see http://sprott.physics.wisc.edu/pickover/esp2.html
