# Supplementary material for: Clinical relevance of appraisals of persistent psychotic experiences in people with and without a need for care: an experimental study
Source: Lancet Psychiatry. 2017 Dec;4(12):927–36. doi: 10.1016/S2215-0366(17)30409-1 (PMC5714590; doi:10.1016/S2215-0366(17)30409-1)
Supplement: Supplementary appendix [file mmc1.pdf]

THE LANCET
Psychiatry

Supplementary appendix

This appendix formed part of the original submission and has been peer reviewed. We post it as supplied by the authors.

Supplement to: Peters E, Ward T, Jackson M, et al. Clinical relevance of appraisals of persistent psychotic experiences in people with and without a need for care: an experimental study. *Lancet Psychiatry* 2017; **4:** 927–36.

APPENDIX

CLINICAL RELEVANCE OF APPRAISALS OF PERSISTENT PSYCHOTIC EXPERIENCES IN PEOPLE WITH AND WITHOUT A NEED FOR CARE: AN EXPERIMENTAL STUDY

SUPPLEMENTARY MATERIALS

Participants

(1) Clinical group:
This group consisted of 84 patients with current positive symptoms (score≥2 ('occasional') on at least one item of the Scale for the Assessment of Positive Symptoms (SAPS)[1], and International Classification of Diseases, 10th revision (ICD-10)[2] F20-39 diagnoses. Participants were recruited from SLaM and the Betsi Cadwaladr University Health Board. Participants who had been exposed to NICE-adherent Cognitive Behaviour Therapy for psychosis (i.e. 6+ months of therapy and/or a minimum of 16 planned sessions) were excluded, since therapy may have impacted on appraisals of experiences being measured.

(2) Non-clinical group:
This group comprised 92 individuals with enduring psychotic experiences who had never been diagnosed with, or treated for, a psychotic disorder. The majority were recruited using our previous sampling strategy[3-7] targeting specialist sources such as psychic and spiritualist fora, mediums, and other special interest websites (see[8] for further details). Individuals were invited to participate if they (i) reported one or more psychotic experience (secondary item) on the Psychosis Screening Questionnaire (PSQ[9]), and 'occasional' (at least monthly) experiences of any positive and Schneiderian first-rank symptom on the Unusual Experiences Screening Questionnaire (UESQ), within the last month and in the absence of drug use/clear consciousness; (ii) whose experiences started more than 5 years previously (to avoid including individuals who may be prodromal); (iii) who did not score 2 ('unmet need') on items covering basic self-care and the 'psychological distress' dimension (in relation to their psychotic experiences) of the Camberwell Assessment of Need Short Appraisal Schedule (CANSAS[10]); (iv) had never been in contact with mental health services/general practitioner in relation to their psychotic experiences (nor had someone else on their behalf); (v) had never been in contact with secondary mental health care; (vi) were judged by the research team to not be in need of care.

Only individuals who scored≥2 ('occasional') on at least one item of the SAPS[1] at the time of recruitment were included. People who had received diagnoses of, and/or treatment for, common mental health problems (such as anxiety and depression), or had been in contact with primary care services for issues unrelated to their psychotic experiences, were included in the study (N=16; 17.4%).

(3) Control group:
This group included 83 individuals with no psychotic experiences (endorsed no items on UESQ and PSQ[9]) and scoring≤1 standard deviation (SD) of the Unusual Experiences (UnEx) sub-scale mean of the Oxford-Liverpool Inventory of feelings and Experiences (O-LIFE[9,11]). They were recruited from community settings or volunteered by non- clinical participants. People who had received diagnoses of, and/or treatment for, common mental health problems, but not those who had been in contact with secondary mental health care, were included in the study (N=5; 6.0%).

Group differences

The groups did not differ in age ($F_{2,256}$)=2.5, p=.09), but there were more men in the clinical than in the other two groups (χ^2=31.3, d.f.=2, p<.001). There were also differences between the groups on ethnicity (White vs. others: χ^2=20.1, d.f.=2, p<0.001), number of years in education ($F_{2,254}$=6.3, p=0.002), and IQ ($F_{2,247}$=71.1, p<0.001): the non-clinical participants were less likely to belong to a British Minority Ethnic group, they were more educated, and had a higher estimated IQ than the non-clinical group, as is typical for these samples[3,4,8]. The controls were selected to be matched to the non-clinical participants on age, gender, ethnicity and education, and therefore did not differ from each other on those variables. The three groups also differed in how they described themselves in terms of spirituality (χ^2=54.2; d.f.=2 p<.001) and religion (χ^2=68.2; d.f.=4 p<.001). A greater proportion of the non-clinical group reported being spiritual and following non-traditional

religions than both the clinical and control groups. The clinical group was more likely to follow a mainstream religion than both the non-clinical and control groups, and were more spiritual than the control group.

The non-clinical group had a younger age of onset of their psychotic experiences than the clinical group ($t_{(174)}$=3.9, p<0.001), and had lived with their experiences for longer ($t_{(174)}$=5.1, p<0.001). The clinical and non-clinical groups did not differ significantly from each other on either the Appraisals of Anomalous Experiences interview (AANEX[3]) current ($F_{1,172}$=2.9, p=0.475[1])) or lifetime ($F_{1,172}$=2.8, p=0.514 total scores, or lifetime presence of auditory hallucinations (X^2=3.6, d.f.=1, p=0.06), which was >75% in both groups. There were some differences between the groups in terms of individual AANEX (current) factor scores: the clinical group scored higher than the non- clinical group on 'Cognitive-Attentional' ($F_{1,172}$=28.4, p<0.007) and 'Dissociative-Perceptual' ($F_{1,172}$=7.5, p=0.048) factors, while the reverse was found for the 'Paranormal-Hallucinatory' factor ($F_{1,172}$=9.3, p=0.021). There were no differences on the two remaining factors, 'Meaning-Reference' ($F_{1,172}$=0.7, p=0.975) and 'First- Rank Symptoms' ($F_{1,172}$=2.8, p=0.507).

A fuller description of the clinical, sociodemographic, and psychological differences between the groups are reported elsewhere[8].

Screening, cognitive assessment and clinical measures

Screening tools
Screening tools were not routinely administered to the clinical group, who were screened through clinicians and/or case-note review. The CANSAS was administered to the non-clinical group only, and the O-LIFE to the control group only.

Unusual Experiences Screening Questionnaire (UESQ)
This screening tool consists of nine items derived from the AANEX-Inventory[3], assessing the presence of a range of positive and Schneiderian First Rank Symptom (such as hallucinations, thought interference, delusional perception), within the last month, in the absence of drug use and in clear consciousness.

Psychosis Screening Questionnaire (PSQ)[9]
The PSQ is an interviewer administered questionnaire that assesses psychotic experiences in the preceding year and comprises five sections covering hypomania, thought disorder, paranoia, strange experiences and hallucinations. Each section has an initial probe, followed by secondary questions that are designed to establish the psychotic quality of experiences. The PSQ has been validated in two national surveys in the UK[12,13]. As we were specifically interested in psychotic experiences, items on hypomania were discarded. The PSQ and UESQ were merged to avoid repetition of items.

Camberwell Assessment of Need Short Appraisal Schedule (CANSAS)[10]
The CANSAS is a comprehensive assessment of clinical and social needs. Only items 1-4 (relating to accommodation, food, home, and self-care), and item 9 (psychological distress in relation to unusual experiences) were used. Scores range from 0-2 ('0' = no problem; '1' = met need; '2' = unmet need).

The Oxford-Liverpool Inventory of Feelings and Experiences (O-LIFE)[11]
Only the Unusual Experiences (UnEx) subscale of this standardised schizotypy questionnaire was used. UnEx includes 30 items describing perceptual aberrations, magical thinking, and hallucinations, and is phenomenologically related to positive symptoms of psychosis. Items are scored Yes or No, with a potential range of scores 0-30. O-LIFE norms[11] indicate a mean of 8.8, and SD=6.2 (therefore cut-off score for this study=15).

Cognitive assessment
Wechsler Adult Intelligence Scale 3rd Edition – Short Form (WAIS-III[14])

A short form was used consisting of one subtest of each cognitive index: Information (Verbal Comprehension), Block Design (Perceptual Organisation), Arithmetic (Working Memory), and Digit Symbol (Processing Speed). The four subtest scaled scores were summed and divided by the total number of subtests (11) to generate a WAIS estimation total score, which was then converted into an Estimated IQ score.

[1] #: All AANEX comparisons were subjected to Sidak adjusted p value = 1-(1- unadjusted p)^n; n = 7

Clinical measures

Clinical assessments were administered to the clinical and non-clinical groups only.

Appraisals of Anomalous Experiences Interview (AANEX-Inventory)[3]*:*

This semi-structured interview was used to elicit participants' current psychotic experiences and their associated emotional and cognitive correlates. The first part of the interview (AANEX-Inventory; short form[15]) consists of 17 anomalous experiences that are rated for both the presence and severity of each experience in the person's lifetime, and currently (within the last month). Each item is rated on a 3-point scale (1=not present; 2=unclear; 3=present). Possible total scores range from 17-51 for both lifetime and current experiences. Five factor scores are also generated via summation of individual item scores[16]: Meaning-Reference (MeanRef ; range 4-12), which reflects manic or hypomanic states and experiences, ideas of reference, 'insight' and prominent 'revelatory' experiences; Paranormal-Hallucinatory (ParaHall; range 3-9), which reflects alterations in sense of agency and passivity, somatic hallucinations, and paranormal experiences such as mediumship, clairvoyance and magic, and perception of other entities/energies; Cognitive-Attention (CogAtt; range 3-9), which reflects non-specific subjective changes or deficits in thinking and attention, such as thought-blockages and loss of automatic skills; Dissociative-Perceptual (DissPer; range 3-9), which reflects dissociative experiences such as depersonalisation and derealisation, along with other global perceptual changes; and First-Rank Symptoms (FRS; range 4-12), which includes auditory hallucinations, experiences of weakened boundaries between self and other such as thought transmission, receptivity, and 'made' emotions.

Scale for the Assessment of Positive Symptoms (SAPS)[1] *and Scale for the Assessment of Negative Symptoms (SANS)*[17]

These interviews are well-established, standardised scales assessing the positive and negative psychosis symptoms. The SAPS consists of 35 items subdivided into four sections: hallucinations, delusions, bizarre behaviour, and positive formal thought disorder. The SANS consists of 25 items subdivided into five sections: affective flattening or blunting, alogia, avolition-apathy, anhedonia-asociality, and attention. Scores for each item reflect level of severity and frequency, and range from '0' (None) to '5' (Severe). Each subscale produces a global rating (also 0-5). The total range of scores for SAPS is 0-175, and for SANS 0-125.

Inter-rater reliabilities

Interviews were audio-recorded for scoring, with the participant's consent. Inter-rater reliabilities (ascertained using 35 interviews rated by the study coordinator (TW) and the individual Research Workers (RWs): London site=15; Bangor site=20; Clinical group=16; Non-Clinical group=19) for the AANEX-Inventory[3] indicated almost perfect agreement (Intra-Class Correlation (ICC)>.8): total number of experiences endorsed (ICC=.995); current experiences (ICC=.997); lifetime experiences (ICC=.998). Inter-rater reliability for the combined Scale for the Assessment of Positive Symptoms (SAPS)[1] & Scale for the Assessment of Negative Symptoms (SANS)[17] also indicated almost perfect agreement (ICC=.904).

For the eight categories, and five dimensions, of appraisals on the AANEX-CAR[3], weighted Kappas were all in the 'substantial agreement' (>0.6) or 'almost perfect' (>0.8) ranges, apart from one category ('psychological') and one dimension ('agency'), which showed 'fair-moderate' agreement (0.4-0.6). The 'psychological' category nevertheless demonstrated 83.9% agreement and so was kept in the analyses, but the 'agency' dimension only showed 67.1% agreement and was therefore dropped from further analyses.

References

1. Andreasen N. Scale for the Assessment of Positive Symptoms (SAPS). 1984.
2. World Health Organisation. ICD-10 Classifications of Mental and Behavioural Disorder: Clinical Descriptions and Diagnostic Guidelines. Geneva: World Health Organisation; 1992.
3. Brett CMC, Peters EP, Johns LC, Tabraham P, Valmaggia LR, McGuire P. Appraisals of Anomalous Experiences Interview (AANEX): a multidimensional measure of psychological responses to anomalies associated with psychosis. *Brit J Psychiat* 2007; **191**:S23-S30.
4. Ward T, Gaynor K, Hunter M, Woodruff P, Garety P, Peters E. Appraisals and responses to experimental symptom analogues in clinical and nonclinical individuals with psychotic experiences. *Schizophrenia Bulletin* 2014; **40**(4): 845-55.
5. Marks EM, Steel C, Peters ER. Intrusions in trauma and psychosis: information processing and phenomenology. *Psychol Med* 2012; **42**(11): 2313-23.
6. Gaynor K, Ward T, Garety P, Peters E. The role of safety-seeking behaviours in maintaining threat appraisals in psychosis. *Behav Res Ther* 2013; **51**(2): 75-81.
7. Heriot-Maitland C, Knight M, Peters E. A qualitative comparison of psychotic-like phenomena in clinical and non-clinical populations. *Brit J Clin Psychol* 2012; **51**: 37-53.
8. Peters E, Ward T, Jackson M, et al. Clinical, socio-demographic and psychological characteristics in individuals with persistent psychotic experiences with and without a "need for care". *World Psychiatry* 2016; **15**(1): 41-52.
9. Bebbington P, Nayani T. The Psychosis Screening Questionnaire. *Int J Method Psych* 1995; **5**(1): 11-9.
10. Slade M, Loftus L, Phelan M, Thornicroft G, Wykes T. The Camberwell Assessment of Need. London: Gaskell; 1999.
11. Mason O, Claridge G. The Oxford-Liverpool Inventory of Feelings and Experiences (O-LIFE): Further description and extended norms. *Schizophr Res* 2006; **82**(2-3): 203-11.
12. Naroo JY. Ethnicity and Mental Health: Findings from a National Community Survey. London: Policy Studies Institute; 1997.
13. Singleton N, Bumpstead R, O'Brien M, Lee A, Meltzer H. Psychiatric Morbidity Among Adults Living in Private Households 2000. London: The Stationery Office; 2001.
14. Wechsler D. Wechsler Adult Intelligence Scale - Third Edition. San Antonio, TX: The Psychological Corporation; 1997.
15. Lovatt A, Mason O, Brett C, Peters E. Psychotic-Like Experiences, Appraisals, and Trauma. *J Nerv Ment Dis* 2010; **198**(11): 813-9.
16. Brett CM, Peters ER, McGuire PK. Which psychotic experiences are associated with a need for clinical care? *European psychiatry : the journal of the Association of European Psychiatrists* 2015.
17. Andreasen N. Scale for the Assessment of Negative Symptoms (SANS). 1984.
